# The Severity of DSS-Induced Colitis Is Independent of the SCFA-FFAR2/3-GLP-1 Pathway Despite SCFAs Inducing GLP-1 Secretion via FFAR2/3

**DOI:** 10.3390/metabo14070395

**Published:** 2024-07-20

**Authors:** Jenna Elizabeth Hunt, Charlotte Bayer Christiansen, Mohammad Yassin, Bolette Hartmann, Stefan Offermanns, Lars Ove Dragsted, Jens Juul Holst, Hannelouise Kissow

**Affiliations:** 1 Department of Biomedical Sciences, Faculty of Health and Medical Sciences, University of Copenhagen, 2200 Copenhagen, Denmark; jenna.hunt@sund.ku.dk (J.E.H.); cbchristiansen@sund.ku.dk (C.B.C.); bhartmann@sund.ku.dk (B.H.); 2 Department of Cellular and Molecular Medicine, Faculty of Health and Medical Sciences, University of Copenhagen, 2200 Copenhagen, Denmark; yassin@sund.ku.dk; 3 Max Planck Institute for Heart and Lung Research, D-61231 Bad Nauheim, Germany; stefan.offermanns@mpi-bn.mpg.de; 4 Department of Nutrition, Exercise and Sports, Faculty of Science, University of Copenhagen, 2200 Copenhagen, Denmark; ldra@nexs.ku.dk; 5 Novo Nordisk Foundation Center for Basic Metabolic Research and Department of Biomedical Sciences, Faculty of Health and Medical Sciences, University of Copenhagen, 2200 Copenhagen, Denmark; jjholst@sund.ku.dk

**Keywords:** SCFA, colitis, mice, GLP-1, fiber

## Abstract

Short-chain fatty acids (SCFAs) are the major microbial metabolites produced from the fermentation of dietary fiber in the gut. They are recognised as secretagogues of the glucagon-like peptides, GLP-1 and GLP-2, likely mediated by the activation of free fatty acid receptors 2 and 3 (FFAR2 and 3) expressed on enteroendocrine L-cells. Fiber-deficient diets are associated with decreased intestinal function and decreased colonic GLP-1 and GLP-2 content. Here, we speculated that the lowered colonic GLP-1 observed following a fiber-free diet was a consequence of decreased SCFA production and a subsequent decrease in FFAR2/3 activation. Furthermore, we explored the consequences of a fiber-free diet followed by intestinal injury, and we mechanistically explored the SCFA-FFAR2/3-GLP-1 pathway to explain the increased severity. Colonic luminal content from mice fed either a fiber-free or chow diet were analysed for SCFA content by LC–MS. FFAR2/3 receptor contributions to SCFA-mediated colonic GLP-1 secretion were assessed in isolated perfused preparations of the colon from FFAR2/3 double knockout (KO) and wild-type (WT) mice. Colitis was induced by the delivery of 3% dextran sulfate sodium (DSS) for 4 days in the drinking water of mice exposed to a fiber-free diet for 21 days. Colitis was induced by the delivery of 3% DSS for 7 days in FFAR2/3 KO mice. The removal of dietary fiber significantly decreased SCFA concentrations in the luminal contents of fiber-free fed mice compared to chow-fed mice. In the perfused colon, luminal SCFAs significantly increased colonic GLP-1 secretion in WT mice but not in FFAR2/3 KO mice. In the DSS-induced colitis model, the removal of dietary fiber increased the severity and prevented the recovery from intestinal injury. Additionally, colitis severity was similar in FFAR2/3 KO and WT mice after DSS application. In conclusion, the results confirm that the removal of dietary fiber is sufficient to decrease the colonic concentrations of SCFAs. Additionally, we show that a fiber-free diet predisposes the colon to increased intestinal injury, but this effect is independent of FFAR2 and FFAR3 signalling; therefore, it is unlikely that a fiber-free diet induces a decrease in luminal SCFAs and sensitivity to intestinal disease involves the SCFA-FFAR2/3-GLP-1 pathway.

## 1. Introduction

The intestinal mucosa plays a crucial role in the absorption of nutrients and maintenance of gut barrier function, both of which are essential for survival [1]. It has been demonstrated that nutrients, particularly fiber, can influence the growth of the intestinal mucosa and improve intestinal functioning. Previous studies employing different fiber sources supplemented in the diets of multiple animal species have shown a positive influence on intestinal function by increasing intestinal mass [2,3,4], mucosal weight [5], crypt depth [2,3], and the expression of tight junction proteins [6]. In agreement, we have previously shown that mice fed a fiber-deficient diet for 21 days had a loss of intestinal mass, decreased levels of glucagon-like peptide-1 and -2 (GLP-1 and GLP-2) in the colon, and increased intestinal permeability after an extended feeding period of 112 days [7]. Other studies have reported that fiber-deficient diets are associated with degradation of the colonic mucus barrier and increased pathogen susceptibility [8,9]. Collectively, these findings suggest that dietary fiber may be an important factor for consideration in the treatment of intestinal diseases characterised by mucosal injury.

Short-chain fatty acids (SCFAs) are a subset of saturated fatty acids with aliphatic chains of less than six carbons. They are predominantly produced by the fermentation of dietary fiber under anaerobic conditions in the human colon [10,11]. The most abundant intestinal SCFAs are acetate (C2), propionate (C3), and butyrate (C4), with concentrations ranging from 50 to 100 mM in the colonic lumen [12,13]. SCFAs are capable of crossing the epithelium [14,15] and may enter the systemic circulation via monocarboxylate transporters, resulting in plasma concentrations in the micromolar range [12]. However, butyrate is a preferred nutrient source for colonocytes [16] and is largely consumed locally. Propionate is metabolised in the liver (although there is great variation between species), decreasing its plasma concentration and making acetate the most abundant SCFA in the circulation [12]. All SCFAs can trigger downstream signalling cascades by activating free fatty acid receptor (FFAR) 2 and FFAR3 [17,18,19]. These receptors share 40% amino acid sequence similarity and are highly conserved in several species of mammals, including mice [18,19]. Both receptors can be activated by two- to five-carbon SCFAs [17], and several groups have confirmed their expression on GLP-1-secreting L-cells at the mRNA [20] and protein levels [21].

SCFAs, particularly propionate, have been demonstrated to be potent secretagogues of GLP-1 in primary cultured colonocytes from humans [22] and in mice [20,23]. Accumulating evidence suggests that SCFA-stimulated GLP-1 secretion involves the activation of the FFAR2 receptor [20,23,24,25], but the downstream signalling mechanisms of GLP-1 release remain disputed [23]. Previously, GLP-2 has been associated with recovery following intestinal injury; however, recent studies have also investigated the potential role of GLP-1 in this process. Indeed, treatment with GLP-1 and GLP-2 analogues has been shown to ameliorate acute small intestinal injury in rodents, while the secretion of both hormones is increased during recovery [26]. Furthermore, L-cell depletion in mice exacerbates intestinal damage and delays recovery following chemotherapy [26].

Here, we aimed to investigate how the removal of fiber from the diet effects colonic luminal SCFA content and the potential impact this could have on SCFA receptors (FFAR2 and FFAR3) and subsequent GLP-1 secretion. Furthermore, we hypothesised that in scenarios of reduced SCFA signalling, such as during removal of fiber from the diet, GLP-1 secretion would also be reduced, thereby increasing the severity and prolonging the recovery from intestinal injury.

## 2. Materials and Methods

Animals. All of the experiments were conducted with permission from the Danish Animal Experiments Inspectorate (licence no. 2018-15-0201-01397). Female C57BL/6JRj mice were purchased from Janvier Laboratories (Saint-Berthevin Cedex, France). The FFAR2/3 double-deficient mouse line (Ffar2−/−;Ffar3−/−) (FFAR2/3 KO) was bred in-house and has been described previously [27]. Heterozygous breeding pairs were used to generate wild-type (Ffar2+/+;Ffar3+/+) (WT) littermates as controls. All mice were housed in individually ventilated cages with 12:12 h light/dark cycles. All mice were acclimatised for one week in the animal facility before experimentation began, and during that time, all mice had ad libitum access to standard chow (Altromin, Lage, Germany, cat. no. 1310) and water.

Fiber-free diet. Colonic luminal content was collected in a previous experiment exploring fiber-free diet and intestinal GLP-1 content. In short, mice were randomly allocated to feeding groups and placed in new housing cages, with four mice per cage. Mice were fed standard chow diet (Altromin, Lage, Germany, cat. no. 1310) or were switched to a fiber-deficient diet (Altromin, Lage, Germany, cat. no. 1013), henceforth referred to as “fiber-free”, for 21 days (Table 1; for the full nutritional composition, see Supplementary Table S1).

Luminal SCFA measurement. Eight-week-old female mice fed either a fiber-free diet or a chow diet (*n* = 8) for 21 days were euthanised by cervical dislocation. The colon was resected and flushed with 0.5 mL of saline, and the contents were collected and stored at −80 °C until further analysis. The luminal contents were manually homogenised, and 50 mg of the homogenate was mixed with 10 μL of 200 mM 3-nitrophenylhydrazine in 50% ethanol and 120 μL of N-(3-dimethylaminopropyl)-N’-ethylcarbodiimide and 6% pyridine in 50% ethanol and incubated for 30 min at room temperature. The solution was then diluted to a volume of 100 μL with 10% ethanol, and 100 μL of internal standard was added. The internal standard was prepared beforehand by derivatising a 50 μL solution containing 20 mM acetic acid, 10 mM propionic acid, 5 mM butyric acid, and 1 mg 13C6-3-nitrophenylhydrazine hydrochloride in 50% ethanol with 25 μL of 120 mM N-(3-dimethylaminopropyl)-N’-ethylcarbodiimide and 25 μL of 6% pyridine in 50% ethanol. A standard curve of serially diluted external standards was prepared from 0.195 μM to 50 μM, and an assay blank (50% ethanol) was treated the same as the luminal samples. The analytes were randomised, and 5 μL was injected into an ACQUITY UPLC system coupled to a quadrupole time-of-flight mass spectrometer (Waters, Milford, USA). The concentrations were determined using QuanLynx software. The analytes were separated on an ACQUITY UPLC BEH C18 VanGuard precolumn (5 mm length, 2.1 mm internal diameter, and 1.7 μm particle size) and an ACQUITY UPLC BEH C18 column (100 mm length, 2.1 mm internal diameter, and 1.7 μm particle size) (Waters, Milford, USA) at 40 °C. The mobile phase was composed of a gradient of acetonitrile ranging from 20 to 100% containing 0.01% formic acid. The relative standard deviation was 15%, and the LLOQ was defined as the lowest measured concentration of 0.0955 μM after extrapolation from the standard curve.

Mouse colon perfusion. Male and female 20- to 30-week-old FFAR2/3 knockout (KO) mice (*n* = 4, 2 males, 2 females) and WT mice (*n* = 4, 2 males, 2 females) were used for colon perfusions. After anaesthesia, a midline incision was made in the abdominal cavity. The arterial supply to the small intestine, cecum, spleen, stomach, and kidneys was ligated. The colon was divided immediately after the caecum and before the entry of the inferior mesenteric artery, leaving a segment for perfusion of an average length of 5.3 ± 0.3 cm. Tubing was inserted into the proximal lumen, and prewarmed saline (0.9% NaCl solution) was used to flush the colon. A catheter was placed into the abdominal aorta, and perfusion was started. A drainage catheter was then placed in the vena portae. The mouse was then euthanised by bilateral incisions of the diaphragm. The colon was subsequently perfused in situ using dedicated rodent organ perfusion equipment (Hugo Sachs Elektronik, March-Hugstetten, Germany). A diagram and detailed description of the perfusion system can be found in Supplementary Figure S1. The perfusion buffer used was modified Krebs–Ringer bicarbonate buffer (5% dextran T-70, 0.1% bovine serum albumin, 10 µM 3-isobutyl-1-methylxanthine (IBMX), 3.5 mM glucose, and 5 mM pyruvate, fumarate, and glutamate). The buffer was heated to 37 °C and gassed for the duration of the experiment with 95% O_2_ and 5% CO_2_. The experiments were preceded by a 30 min washing period to stabilise hormone secretion. The vascular perfusion flow rate was maintained at 1.5 mL/min, and the luminal flow rate was 0.025 mL/min. A 100 mM SCFA mixture (52 mM acetate, 30 mM butyrate, and 18 mM propionate) was infused luminally for 15 min at an initial flow rate of 0.1 mL/min for the first 3 min and then at 0.025 mL/min thereafter. Following stimulation, saline was infused at an initial flow rate of 0.1 mL/min for the first 3 min and then 0.025 mL/min for the next 12 min. Then, a 1 mM SCFA mixture was infused via the vasculature for 10 min at a flow rate of 0.075 mL/min. At the end of the experiment, 10 mM bombesin (BSS) was infused as a positive control. The effluent samples were collected by a fraction collector each minute, and the samples were stored on ice and subsequently stored at −20 °C until analysis. Total GLP-1 was measured using a radioimmunoassay specific for the C-terminus of the GLP-1 molecule (antibody code no 89390), which reacted equally to intact GLP-1 and the primary (N-terminally truncated) metabolite. The assay showed no cross-reaction with related peptides and had a dynamic range of 1–320 pmol/l. The assay sensitivity was less than 1 pmol/l, and the intra-assay coefficient of variation was less than 10%.

DSS-induced colitis. Dextran sulfate sodium (DSS) (molecular weight 40,000) was prepared at a concentration of 3% in drinking water. Eight-week-old female mice fed either a fiber-free diet or a chow diet for 21 days received DSS ad libitum for 4 days and were then provided with regular drinking water until the mice were euthanised either on day 6 (*n* = 8) or day 13 (*n* = 8). Female and male FFAR2/3 KO mice and wild-type littermates, 8 to 12 weeks of age, received 3% DSS ad libitum for 7 days and were then provided with regular drinking water until the mice were euthanised either on day 7 (*n* = 9–12) or day 14 (*n* = 9–14). The control groups (not receiving DSS) received regular drinking water from the same source throughout. Water and food consumption was recorded daily. The general health of the mice was assessed daily by monitoring body weight (BW) and observing clinical progression. To alleviate suffering, mice with severe BW loss were euthanised. The daily disease activity index (DAI) was calculated based on a system scoring BW loss (0 points, <1%; 1 point, 1–5%; 2 points, 5–10%; 3 points, 10–20%; and 4 points, >20%) and stool consistency (0 points, well-formed faeces; 2 points, semi-formed stools; 4 points, diarrhoea; and 6 points, bloody diarrhoea). At the end of the experiment, the mice were euthanised by cervical dislocation. The colon was resected, measured, flushed with saline, and weighed. A 2 cm piece of the distal colon was removed, fixed in 10% neutral formalin buffer for 24 h, and subsequently transferred to 70% ethanol until further processing for histology.

Histological scoring. Fixed colons were longitudinally embedded in paraffin blocks. The block was trimmed to expose the lumen and subsequently the colonic crypts and sectioned (4 µm). Sections were stained with haematoxylin and eosin. Histological scoring was performed by an experienced histologist using high-quality virtual slides created using a Zeiss Axio Scan Z1 slide scanner (Carl-Zeiss, Oberkochen, Germany). The observer was blinded to the origin of the sections. Scoring ranged from 0 to 6 by adding the tissue damage score (0, no mucosal damage; 1, lymphoepithelial lesions; 2, surface mucosal erosion; and 3, extensive mucosal damage, extension into the deeper structure) to the inflammatory cell infiltration score (0, occasional cell infiltrate; 1, increased number of infiltrating cells; 2, inflammatory cells extending to the submucosa; and 3, transmural extension of the inflammatory cells).

Statistics. All of the statistical analyses were performed using GraphPad Prism 9 (GraphPad, La Jolla, CA, USA). All of the data are expressed as the mean ± SEM. The concentrations of SCFAs were compared by an unpaired *t*-test. TheGLP-1 secretion from colon perfusions is presented as the total output (fmol/min; effluent concentration × perfusion flow). Statistical analysis of the treatments was performed by comparing the mean basal output with the mean output during the stimulation using a paired *t*-test. For the basal values, values obtained 5 min prior to the administration of SCFAs, luminally or vascularly, were used. For luminal stimulation, values obtained after 10 min of observation, starting 5 min into the infusion, were used; for vascular infusions, values obtained after 10 min of observation were used. Repeated measures (BW and DAI) were analysed using mixed-effects two-way ANOVA followed by Bonferroni’s multiple comparisons test. Survival curves were compared using the Kaplan–Meier method, and groups were compared using the log-rank (Mantel–Cox) test. Otherwise, the data were compared using one-way ANOVA followed by Bonferroni’s multiple comparisons test. Differences were considered significant at *p* < 0.05. The number of animals included in each experiment was based on power calculations using endpoint standard deviations known from previous experiments. A power of 80% and an alpha of 0.05 were chosen.

## 3. Results

### 3.1. The Removal of Dietary Fiber Decreased Colonic Luminal SCFA Levels and Stimulated Colonic GLP-1 Secretion

**Figure 1:**
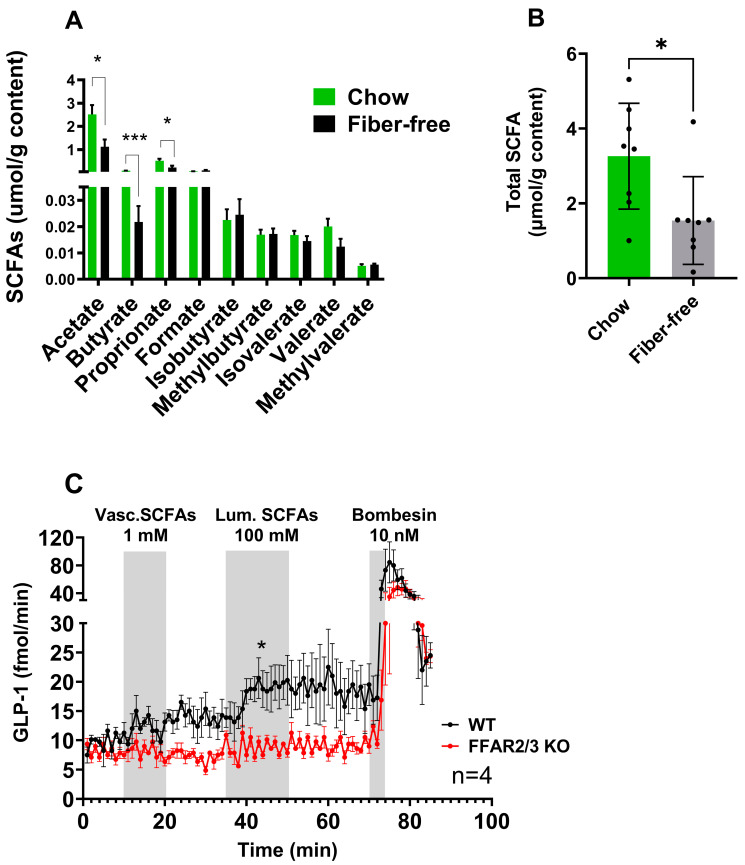
The removal of dietary fiber decreased colonic luminal SCFA levels, which was shown to stimulate colonic GLP-1 secretion. (**A**) SCFA concentrations in luminal contents from chow- and fiber-free diet-fed mice. (**B**) Total SCFA concentrations in luminal contents from chow- and fiber-free diet-fed mice. The data are presented as the mean ± SEM. The concentrations of SCFAs were compared by an unpaired *t*-test. * *p* < 0.05 and *** *p* < 0.001. (**C**) GLP-1 secretion from the isolated perfused colons from FFAR2/3 KO and WT mice stimulated vascularly with 1 mM SCFAs and luminally with 100 mM SFCAs. Bombesin (10 nM) served as a positive control. GLP-1 secretion is presented as the total output (fmol/min; effluent concentration × perfusion flow). A paired *t*-test was used to compare the mean output from each group. * *p* < 0.05.

Twenty-one days of fiber-free feeding decreased the concentrations of acetate (*p* < 0.05), butyrate (*p* < 0.001), and propionate (*p* < 0.05) in the colonic luminal content (Figure 1A). The other measured SCFAs, including branched chain SCFAs, remained unchanged. Overall, the total measured SCFAs decreased (*p* < 0.05) after fiber-free feeding (Figure 1B). Fiber-free mice consumed fewer total calories per day due to a slightly lower food intake. Detailed information of the dietary intake and body weight changes can be found in [7].

The ability of SCFAs and the involvement of FFAR 2 and 3 in the pathway to stimulate colonic GLP-1 secretion were investigated using a mouse perfused colon model and an SCFA mixture (acetate, butyrate, and propionate). Luminal SCFA administration (total concentration 100 mM) resulted in increased GLP-1 secretion in WT mice but not in FFAR2/3 KO mice compared to baseline. Vascular infusions of 1 mM did not increase GLP-1 secretion in either WT or KO mice (Figure 1C). Both male and female mice were used in the experimentation and no sex-related differences were observed.

### 3.2. The Removal of Dietary Fiber Increased DSS-Induced Colitis Severity

To investigate whether the removal of dietary fiber would impact the severity of DSS-induced colitis, mice fed either a fiber-deficient diet or a standard chow diet for 21 days were given 3% DSS in their drinking water for four days. Consistently, throughout the four days of DSS treatment, the chow-fed mice consumed more water than the fiber-free diet-fed mice. The chow + DSS mice consumed more drinking water (and therefore more DSS) than the fiber-free + DSS mice on each of the days (*p* < 0.01) (Supplementary Figure S2A). Compared with chow + water treatment, DSS treatment in the chow + DSS group reduced food intake on days 5 and 6 (*p* < 0.05 and *p* < 0.01) (Supplementary Figure S2B). Similarly, food intake was lower on days 4, 5, and 6 in the fiber-free + DSS group than in the fiber-free + water group (*p* < 0.01, *p* < 0.01, and *p* < 0.01, respectively) (Supplementary Figure S2C).

**Figure 2:**
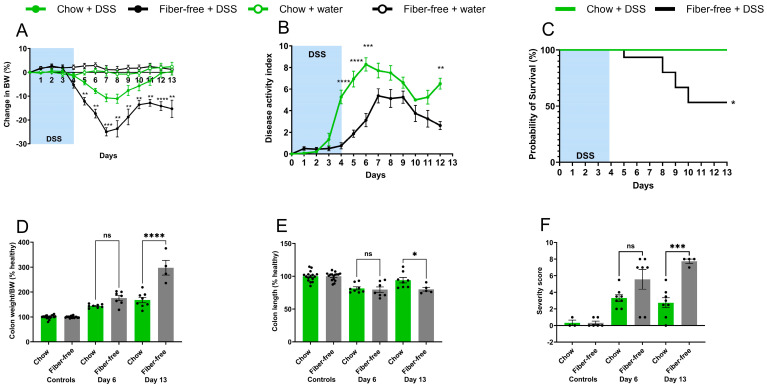
The removal of dietary fiber increased DSS-induced colitis severity and delayed recovery. (**A**) Daily body weight (BW) change; (**B**) disease activity index in DSS-treated mice. The data are shown as the mean ± SEM. Groups in A and B were compared using a two-way ANOVA followed by Bonferroni’s multiple comparisons test. ** *p* < 0.01, *** *p* < 0.001, and **** *p* < 0.0001. (**C**) Survival probability of chow- and fiber-free diet-fed mice after treatment with DSS. Survival curve comparison was performed using the Kaplan–Meier method and groups were compared using a log-rank (Mantel–Cox) test. * *p* < 0.05. (**D**) Colon weight normalised to BW (% of healthy); (**E**) colon length (% of healthy); (**F**) histological severity score. Data are shown as the mean ± SEM. Groups were compared using a one-way ANOVA followed by Bonferroni’s multiple comparisons test. * *p* < 0.05, *** *p* < 0.001, **** *p* < 0.0001, and ns = non-significant.

**Figure 3:**
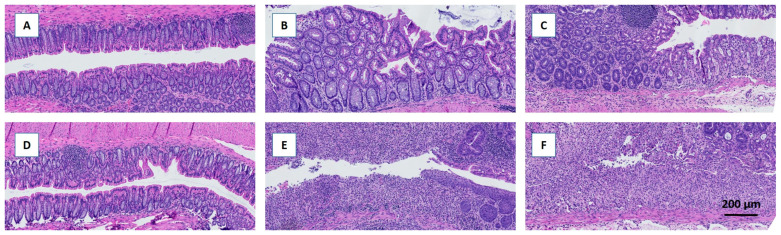
Representative HE-stained histological sections of the colon following DDS-induced colitis. Removal of dietary fiber increased the histopathological severity score, which was most pronounced on day 13. (**A**–**C**) Chow-fed mice. (**D**–**F**) Fiber-free diet-fed mice. (**A**,**D**) Controls. (**B**,**E**) Day 6 after induction of colitis. (**C**,**F**) Day 13 after induction of colitis.

Daily monitoring of BW showed greater decreases immediately after DSS treatment in the fiber-free group than in the chow group; furthermore, these mice were incapable of recovering their BW on day 13, unlike the chow-fed mice (Figure 2A). The DAI was greater in the fiber-free + DSS group than in the chow + DSS group (Figure 2B). There were significant differences between days 4–6 (*p* < 0.0001, *p* < 0.0001, and *p* < 0.001) and day 12 (*p* < 0.01). Furthermore, four mice from the fiber-free + DSS group were sacrificed on days 5, 8, 9, and 10, respectively, due to excessive BW loss. Consequently, mice receiving DSS and the fiber-free diet had a lower percentage of survival than the chow-fed mice (*p* < 0.05) (Figure 2C). Fiber-free feeding decreased the colon weight in the healthy control groups (*p* < 0.0001 in the six-day study and *p* < 0.05 in the 13-day study); therefore, the relative colon weight and colon length are shown as percentages of the control. DSS treatment increased the colonic weight on day 6, with no differences between the feeding groups. On day 13, compared with chow-fed mice, fiber-free diet-fed mice continued to have increased colon weights (*p* < 0.0001) (Figure 2D). DSS treatment shortened the colon length on day 6, with no differences between the feeding groups. On day 13, the fiber-free diet-fed mice had shorter colons than the chow-fed animals (*p* < 0.05) (Figure 2E). Colitis severity was also assessed by scoring distal colonic tissues from histological longitudinal sections. Histopathologic analysis indicated severe gut pathology in mice receiving DSS, with a visible increase in inflammatory cell infiltration and surface mucosal erosion in both feeding groups. In agreement with the DAI and BW results, the removal of fiber had a tendency to increase the severity score (*p* = 0.07) on day 6 and significantly increase the severity score on day 13 (*p* = 0.001) (Figure 2F and Figure 3A–F).

### 3.3. Attenuation of FFAR2/3 Signalling Did Not Affect DSS-Induced Colitis Severity

**Figure 4:**
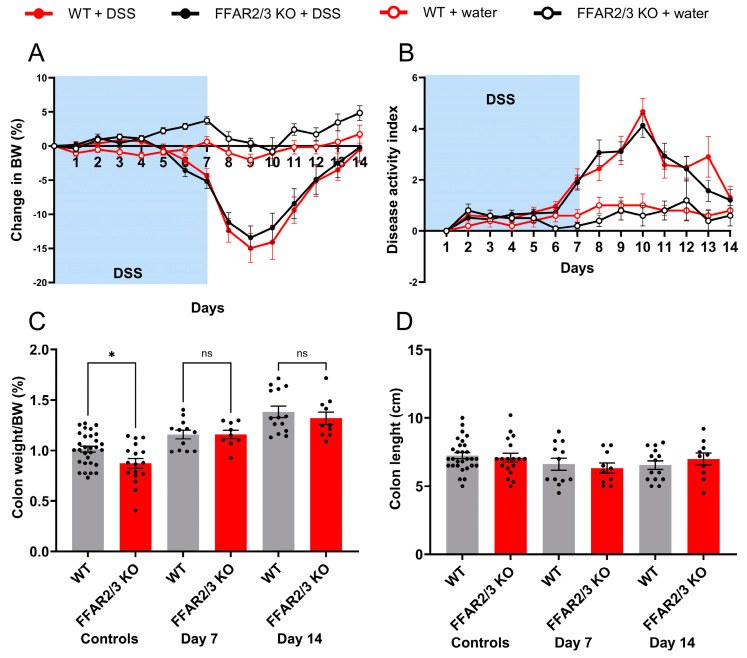
Attenuation of FFAR2/3 signalling did not affect DSS-induced colitis severity. (**A**) Daily body weight (BW) change; (**B**) disease activity index in DSS-treated mice. Data are shown as the mean ± SEM. Groups in A and B were compared using a two-way ANOVA followed by Bonferroni’s multiple comparisons test. No significant differences were found between the DSS-treated groups. (**C**) Colon weight normalised to BW; (**D**) colon length. Data are shown as the mean ± SEM. Groups were compared using a one-way ANOVA followed by Bonferroni’s multiple comparisons test. No significant differences were found between the DSS-treated groups.

To elucidate the involvement of the SCFA receptors, FFAR2 and FFAR3, in DSS-induced colitis, we repeated the DSS experiments in dual FFAR2/3 knockout mice. All animals receiving DSS exhibited decreases in body weight, but there were no significant differences between the genotypes (Figure 4A). Furthermore, disease activity did not differ between genotypes (Figure 4B), and there was no difference in survival. Colon weight increased in all DSS groups on days 7 and 14, but there were no differences between the genotypes (Figure 4C). Colon length decreased in all DSS groups, again with no differences between genotypes (Figure 4D).

## 4. Discussion

In this study, we showed that feeding mice a fiber-deficient diet for 21 days resulted in a significant decrease in the colonic luminal SCFA concentration. Furthermore, the delivery of SCFAs to the colonic lumen at physiological concentrations stimulated GLP-1 secretion. Given these observed outcomes, we hypothesised that a fiber-deficient diet might predispose individuals to increased severity and delayed recovery from intestinal diseases characterised by mucosal injury, where glucagon-like peptides play a pivotal role. We investigated this possibility using a mouse model of DSS-induced colitis, a model that mimics human ulcerative colitis-like pathologies.

SCFAs are recognised as secretagogues of glucagon-like peptides based on multiple experimental approaches, including primary cell culture [20,22,23], isolated colonic perfusion [28], and human trials [29]. Given these observations, and our own previous research showing that a fiber-free diet decreases colonic GLP-1 and GLP-2 content [7], we speculated that the lowered colonic GLP-1 observed following a fiber-free diet was a consequence of decreased SCFA production and the subsequent decrease in FFAR2/3 activation on GLP-1-secreting L-cells. In this study, we observed a decrease in total colonic luminal SCFAs in mice fed a fiber-deficient diet, supporting previous observations that a low-fiber diet decreased cecal SCFA levels in mice, which corresponded with decreased circulating SCFA levels [30]. Despite the fermentation of fibers being the largest source of SCFAs, SCFAs can also be produced from amino acid metabolism [31]. Branched chained SCFAs are the major products of branched-chain amino acid (leucine, isoleucine, and valine) fermentation and are increasingly being investigated for their influence on microbiota composition and human health [32]. Following the removal of fiber, it has been speculated that there could be an increase in proteolytic fermentation [33]. Here, the removal of fiber did not significantly affect the concentrations of the measured branched chained amino acids (isovalerate, methylbutyrate, and isobutyrate), suggesting that branched-chain amino acid fermentation was unlikely increased.

The demonstrated decrease in SCFAs following the removal of dietary fiber may have consequences for the colonic luminal environment, such as decreasing the availability of ligands for the SCFA receptors, FFAR2 and FFAR3, and a subsequent decrease in glucagon-like peptide levels. Using an isolated colon perfusion mouse model and FFAR2/3 KO mice, we investigated the FFAR receptor contributions to GLP-1 secretion upon both luminal and vascular infusions of SCFAs. The SCFA mixture infused luminally reflected a physiological dose, while the dose infused vascularly was supraphysiological [12]. Luminal infusion (100 mM) produced a rapid increase in GLP-1 secretion only in WT mice, therefore confirming the necessity of FFAR2/3 signalling in luminal SCFA-stimulated GLP-1 secretion. Interestingly, vascular infusion (1 mM) did not elicit a response in either WT or KO mice. These results support previous studies of the isolated perfused colon in rats, where 100 mM luminal infusion of an acetate and butyrate mixture stimulated GLP-1 secretion; however, in these studies, 1 mM vascular fluid was also capable of stimulating GLP-1, unlike in our experiments [28]. The differences in vascular outcomes may be due to the different ratios of SCFAs in the mixtures or the presence of propionate; however, propionate has been shown to be a strong GLP-1 secretagogue in primary mouse cultures [20,23].

Next, after showing that a fiber-free diet can decrease the luminal SCFAs demonstrated to be luminal secretagogues of GLP-1, we investigated how pretreatment with a fiber-deficient diet (21 days) impacted both the acute and recovery stages of colonic colitis using a robust DSS-induced colitis mouse model. As hypothesised, the removal of dietary fiber increased the severity and prevented the recovery of DSS-induced colitis. In particular, the removal of fiber acutely increased body weight loss and increased daily disease activity scores. In recovery stage, the removal of fiber also affected colon-specific endpoints, including the colon length and severity score, suggesting the increased importance of fiber in the recovery stage. These results support the observations that supplemented or high-fiber diets ameliorate ulcerative colitis [34,35]. The protective effect of dietary fiber in these studies is thought to be at least partly attributable to the increased production of SCFAs. Similarly, butyrate has been shown to promote epithelial barrier function by exerting anti-inflammatory effects [36], upregulating tight junction proteins [37], and inducing the production of antimicrobial peptides [38]. Furthermore, SCFAs play a role in priming the inflammasome response to promote epithelial integrity [39]. The modulation of cell function by SCFAs may involve inhibiting histone deacetylase activity, thereby altering gene transcription, or by binding to the G protein-coupled receptors, FFAR 2 and FFAR 3. Therefore, it could be speculated that the increased severity of colitis in the fiber-free diet-fed mice could be a consequence of decreased SCFA production and decreased GLP-1 secretion. This is further supported by the observed lower concentration of fecal SCFAs in inflammatory bowel disease patients than in healthy subjects [40] and the clinical benefits of SCFA treatment for colitis [41,42,43].

We investigated whether the SCFA-FFAR2/3-GLP-1 pathway is important for protection against intestinal disease utilising dual-receptor FFAR2/3 KO mice treated with DSS for 7 days and hypothesised that FFAR2/3 KO mice would present with severe colitis. However, DSS treatment decreased body weight, increased colonic weight and DAI, and decreased colonic length independent of FFAR signalling. This finding ruled out the involvement of the SCFA-FFAR2/3-GLP-1 pathway. Nevertheless, our findings do not exclude the possibility that the reduction in GLP-1 due to a lack of fiber is responsible for the reduced protection because endocrine L-cells are activated by numerous substances, e.g., nutrients, bile acids, and other metabolites. For instance, the concentration of indole, a metabolite of tryptophan metabolism, is diminished in the luminal contents of mice fed a diet lacking fiber, and this reduction stimulates GLP-1 in the perfused mouse colon. [44,45]. A dual-receptor knockout mouse was used instead of two single receptor knockout mice since both receptors are encoded at a single chromosomal locus in both mice and humans; therefore, the disruption of one receptor may also affect the other, which has been proposed to explain the inconsistencies in the literature regarding the roles of these FFARs [27].

The study has several limitations. Firstly, the diets differed in energy density, which could have contributed to a difference in colitis severity that was not accounted for. However, comparing diets involving a total lack of one component and then matching it with a control diet with the same energy density without adding other possible variances is impossible. Secondly, since we ruled out the SCFA-FFAR2/3-GLP-1 pathway, we can only speculate on the mechanism whereby a fiber-free diet increases the severity of DSS-induced colitis. Furthermore, we cannot conclude from our experiment that the increased injury after a fiber-free diet is a consequence of reduced endogenous GLP-1. This would require the use of a GLP-1R KO mouse in the DSS model, which would be extremely relevant. Indeed, further studies are needed to determine exactly how fiber protects the intestines and whether GLP-1 is involved in this process. 

In conclusion, the results confirm that the removal of dietary fiber is sufficient to decrease colonic concentrations of SCFAs. Additionally, we show that a fiber-free diet predisposes the colon to increased intestinal injury, but this effect was independent of FFAR2 and FFAR3 signalling. We established that SCFA-mediated GLP-1 secretion requires the presence of FFAR 2/3 signalling; therefore, it is unlikely that a fiber-free diet induces a decrease in luminal SCFAs and sensitivity to intestinal disease involves the SCFA-FFAR2/3-GLP-1 pathway. The results contribute to the understanding of the impact of dietary habits and specific food components on the endocrine function of the colon and may highlight the importance of dietary fiber in the pathobiology of ulcerative colitis, which may help to improve clinical outcomes.

## Figures and Tables

**Table 1:** Nutritional composition of the diets.

Group	Fiber-Free	Chow
Total energy (kcal/kg)	3662	3340
Fat (kcal/kg)	457	463
Protein (kcal/kg)	691	901
Carbohydrate (kcal/kg)	2514	1976
Crude fiber (mg/kg)	1650	45,480

## Data Availability

The original contributions presented in the study are included in the article/Supplementary Material, further inquiries can be directed to the corresponding author.

## Supplementary Materials

The following supporting information can be downloaded at: https://www.mdpi.com/article/10.3390/metabo14070395/s1, Table S1: Nutritional comparison of fiber-free diet and chow. Figure S1: Diagram and detailed description of the perfusion system. Figure S2: Water and diet consumption.
