# Supplementary material for: The Severity of DSS-Induced Colitis Is Independent of the SCFA-FFAR2/3-GLP-1 Pathway Despite SCFAs Inducing GLP-1 Secretion via FFAR2/3
Source: Metabolites. 2024 Jul 20;14(7):395. doi: 10.3390/metabo14070395 (PMC11278623; doi:10.3390/metabo14070395)
Supplement: Supplementary file 1 [file metabolites-14-00395-s001.zip › metabolites-3049827-supplementary.pdf]

Supplementary Materials

Table S1. Nutritional comparison of fiber-free diet and chow.

		**C1013** ***Fiber-Free***	**1310** ***Chow***
Metabolized energy	Fat	457.0 (12%)	463.0 (14%)
(kcal/kg)	Protein	691.0 (19%)	901.0 (27%)
	Carbohydrates	2514.0 (69%)	1976.0 (59%)
Crude nutrients and moisture	Moisture	81,100.0	111,241.0
(mg/kg)	Crude Ash	54,870.0	61,128.0
	Crude Fibre	1650.0	45,480.0
	Crude Fat	50,830.0	51,398.0
	Crude Protein	172,650.0	225,155.0
	Nitrogenfree extractives	638,900.0	505,599.0
Carbohydrates	Monosaccharides	0.0	0.0
(mg/kg)	Disaccharides	137,305.0	54,151.0
	Polysaccharides	471,727.0	350,300.0
Minerals	Calcium	9308.0	7062.0
(mg/kg)	Potassium	7092.0	10,144.0
	Magnesium 2,055	685.0	2055.0
	Sodium 2,154	2486.0	2154.0
	Phosphorus	7529.0	5090.0
Trace elements	Aluminium	2.8	81.9
(mg/kg)	Chlorine	3630.0	3382.9
	Iron	178.5	191.0
	Flourine	4.2	3.1
	Iodine	0.5	1.5
	Cobalt	0.2	0.4
	Copper	5.8	13.9
	Manganese	100.8	77.7
	Molybdenum	0.2	1.5
	Sulfur	2791.5	974.4
	Selenium	0.3	0.3
	Zinc	29.2	85.0
Amino acids	Alanine	2528.0	10,284.0
(mg/kg)	Arginine	9829.0	14,822.0
	Aspartic acid	3583.0	21,735.0
	Cystine	3196.0	3244.0
	Glutaminc acid	23,675.0	43,649.0
	Glycine	3136.0	9565.0
	Histidine	5276.0	5508.0
	Isoleucine	7223.0	9668.0
	Leucine	14,763.0	17,123.0

	Lysine	17,401.0	11,326.0
	Methionine	7223.0	3171.0
	Phenylalanine	7172.0	10,549.0
	Proline	12,763.0	0.0
	Serine	5268.0	0.0
	Threonine	7154.0	0.0
	Tryptophan	1977.0	0.0
	Tyrosine	9285.0	0.0
	Valine	3296.0	0.0
Fatty Acids	Arachidic acid C-20:0	50.0	148.0
(mg/kg)	Eicosanoic acid C-20:1	150.0	185.0
	Alpha-Linolenic acid C-18:3	150.0	3018.0
	Linolenic acid C-18:2	28,500.0	21,996.0
	Palmitic acid C-16:0	2500.0	5342.0
	Stearic acid C-18:0	1350.0	1615.0
	Oleic acid C-18:1	13,500.0	9287.0
Added vitamins			
(IU/kg)	Vitamin A	15,000.0	41,250.0
(IU/kg)	Vitamin D3	500.0	1650.0
(mg/kg)	Vitamin E	180.0	210.0
	Vitamin K3	10.0	8.0
	Vitamin B1	20.0	50.0
	Vitamin B2	20.0	33.0
	Vitamin B6	15.0	25.0
(µg/kg)	Vitamin B12	41.0	66.0
(mg/kg)	Nicotinic acid	50.0	99.0
	Pantothenic acid	50.0	58.0
	Folic acid	10.0	6.0
(µg/kg)	Biotin	201.0	555.0
(mg/kg)	Choline chloride	1012.0	1650.0
	Vitamin C	20.0	99.0

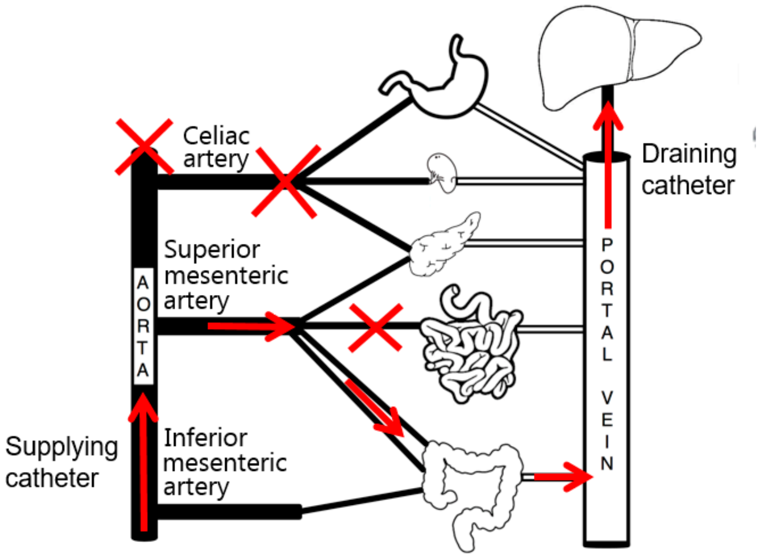

Figure S1. Diagram and detailed description of the perfusion system. The rat colon is perfused in situ after an operation where it is isolated from the remaining circulation by ligating the vascular supplies to other parts of the body (marked with red crosses). A catheter inserted into the aorta establishes perfusion of the colon and a draining catheter inserted into the portal vein ensures collection of the venous effluent. Thus, only the colon is kept artificially alive by mechanically maintaining a constant flow of perfusion medium (1.5 mL/min). To exclude impact from the luminal content the colonic lumen is emptied prior to experiment by gently flushing with saline. After successful operation, the colon is vascularly perfused for a 30 min equilibrium period allowing the organ to adjust to the new condition. Success criteria include adequate vascular perfusion flow, appropriate respiration (O_2 uptake and CO_2 production), constant and appropriate perfusion pressure, preserved histology and, most importantly, preserved responses to the positive control of hormone secretion, bombesin (PLC-activator). The illustration is made by Ida M Modvig.

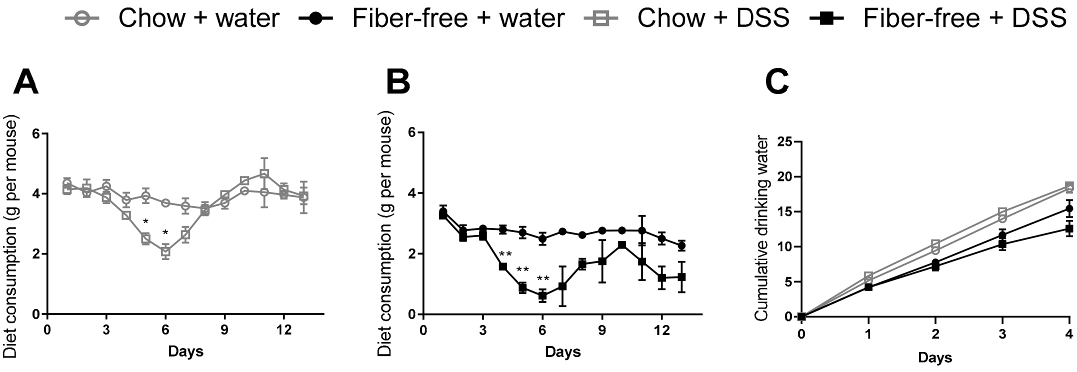

Figure S2. Water and diet consumption. (A) Cumulative water and water + DSS consumption shown as ml per day per mouse. (B) Diet consumption in chow mice groups shown as g per mouse per day. (C) Diet consumption in fiber-free mice. Data are shown as ± SEM. * $p < 0.05$ and ** $p < 0.01$.
